# Research with Older Asian American Family Caregivers Pre- and During the Pandemic: Challenges and Lessons Learned

**DOI:** 10.1093/geroni/igab046.3061

**Published:** 2021-12-17

**Authors:** Jung-Ah Lee, Hannah Nguyen

**Affiliations:** 1 University of California, Irvine, Irvine, California, United States; 2 California State University, Dominguez Hills, Carson, California, United States

## Abstract

Research with hard-to-reach, monolingual adults from ethnic minority communities can present a multitude of challenges throughout the research process. This presentation will highlight challenges and lessons learned from two pilot studies with Vietnamese-, Cambodian-, and Korean-American family caregivers aged 50 and older. The first study (n=9) implemented a one-on-one, telephone-based psychosocial intervention before the COVID-19 pandemic; the second is an ongoing study (n=12) consisting of a group-based intervention via Zoom. Throughout recruitment, the following challenges arose: addressing the lack of familiarity with research among caregivers, earning the trust of caregivers, and identifying creative ways to recruit caregivers to participate. During study implementation, common challenges included: caregivers’ unpredictable daily schedule that made it difficult to participate in the scheduled classes, caregivers feeling apprehensive about technology and Zoom, access to reliable internet, and facilitating participation and engaging the voices of caregivers over the phone or via Zoom. Strategies were identified to address these barriers: engaging the support and collaboration of trusted, bilingual and bicultural community-based providers, building culturally-responsive rapport with caregivers, and seeking continuous feedback from caregivers to improve the appeal of the project implementation. The COVID-19 pandemic added an additional layer of difficulty to the research, requiring creativity and flexibility in implementation that took into consideration caregivers’ heightened anxiety, distress, lack of participation due to around-the-clock care, and loss and grief. The challenges and lessons learned from these studies could guide the development of future research efforts and strategies to effectively engage older hard-to-reach, monolingual Asian American caregivers.

